# Clinical and radiological features of down's arthropathy

**DOI:** 10.1186/1546-0096-12-S1-P30

**Published:** 2014-09-17

**Authors:** Charlene Foley, Emma MacDermott, Orla Killeen

**Affiliations:** 1 National Centre for Paediatric Rheumatology (NCPR), Dublin, Ireland

## Introduction

The ‘Arthropathy of Down syndrome’ was first described in 1984. Three decades on we still have limited literature on the clinical and radiological features of this arthritis, despite the fact that it is thought to be 3-6 times more common than JIA in the general paediatric population. Down’s Arthropathy (DA) is rarely recognised at onset, and remains under-diagnosed and largely under-reported in this population group. Ireland has one of the highest Trisomy 21 birth rates in Europe (1/547), & therefore provides an ideal setting for such a study. **Research Q's - 1.** What are the clinical & radiological features of DA? **2.** Is DA missed, leading to a delay in dx?

## Objectives

To perform a musculoskeletal examination on children with T21, aged 0-18 years & document - 1. Presence of features to suggest old and/or present arthritis. 2. Radiological findings.

## Methods

From May 2013 to September 2014, Children with T21 (aged 0-18years) will be invited to attend a screening clinic. Screening involves completion of a health questionnaire & musculoskeletal examination. Suspected cases of DA will be invited to attend the NCPR for Ix, Rx & F/U as per normal clinical practice.

## Results

To date, 370 children with Trisomy 21 have enrolled in the study, 56% Male. 17 new cases of DA have been detected, only three (17.6%) of which were referred with suspected arthritis. In total, 28 children with DA now attend the NCPR for management of their arthritis, the largest cohort ever reported in the literature. We estimate the Point Prevalence of DA in Ireland to be 17-18/1000. For comparison, the UK Prevalence of JIA is 1-2/1000. *Table*1 compares characteristics of our DA cohort to a JIA comparison gp. Table 1.

**Table 1:** Comparison of study characteristic by diagnosis

Characteristic		DA		JIA		p value
		
		(n=28)		(n=21)		
**Small Joint Involvement**	*Mean (sd)*	4.46 (1.95)		3.05 (2.29)		**p<0.01**

**Time to Diagnosis**	*Mean (sd)*	1.71 (1.47)		0.74 (0.86)		**p<0.05**

**Characteristics**		**n**	**(%)**	**n**	**(%)**	**p value**

**Gender**	*Male*	14	50.0	11	52.4	ns
	
	*Female*	14	50.0	10	47.6	

**Rx MTX**		8	28.5	7	33.3	ns

**MTX Nausea**		6	75.0	1	14.3	**p<0.05**

**Features of DA – Our experience -** Polyarticular RF Neg Presentation (69% of DA cohort). Finger involvement (77% of DA cohort) – significantly greater proportion than seen in the JIA comparison group. Erosive changes noted on X-Ray at presentation (27% of cohort). MTX nausea common, but a good response to steroid intra-articular joint injections observed. General lack of awareness about the increased risk of arthritis in children with T21.

## Conclusion

Children with T21 are at increased risk of developing arthritis, however there is often a delay in diagnosis. Reasons for this are multifactorial. Early Dx & Rx of DA is key to preventing irreversible joint destruction & long-term functional impairment. MTX nausea is a significant barrier to successful treatment of DA with this DMARD. However, a good response to steroid joint injections has been observed. We advocate that children with T21 have an annual musculoskeletal assessment as part of their Health Screening Programme.

## Disclosure of interest

None declared.

